# Risk factors and prevalence of diabetic peripheral neuropathy: A study of type 2 diabetic outpatients in Bangladesh

**DOI:** 10.4103/0973-3930.60004

**Published:** 2010

**Authors:** Kjersti Mørkrid, Liaquat Ali, Akhtar Hussain

**Affiliations:** Institute of General Practice and Community Medicine, Department of International Health, University of Oslo. Postbox 1130 Blindern, 0318 Oslo, Norway; 1 Bangladesh Institute of Research and Rehabilitation in Diabetes, Endocrine and Metabolic Disorders (BIRDEM), Dhaka, Bangladesh

**Keywords:** Peripheral neuropathy, risk factors, type 2 diabetes

## Abstract

**Aims/Hypothesis:**

The purpose of the study is to estimate the prevalence and risk factors for diabetic peripheral neuropathy (DPN) in type 2 diabetic outpatients at the BIRDEM hospital, Bangladesh.

**Materials and Methods:**

Type 2 diabetic outpatients, diagnosed 5-11 years prior to the investigation were randomly selected for the study. DPN was assessed using the Neuropathy Symptom Score (NSS) and Neuropathy Disability Score (NDS). Data about demographics, blood pressure, height, weight, waist and hip circumference, and random blood and urine samples were collected.

**Results:**

Two hundred and ninety four (139 men, 155 women) type 2 diabetic outpatients were studied. The overall DPN prevalence was 19.7 %; male (20.9%), female (18.7 %). The prevalence increased with age (from 11.1% in the 23-40 year-old group to 32.3% in the 60-80 year-old group) and duration of diabetes (from 14.1% in patients with five years to 29.2% in patients with 9-11 years duration). Age > 60 years (OR 4.2, 95% CI 1.4-12.3), low/normal WHR (OR 3.8, 95% CI 1.6-9.3), income < 800 TK (OR 3.1, 95% CI 1.1-9.3) and insulin treatment (OR 2.0, 95% CI 1.0-4.0) were independent, significant risk factors. Longer duration of diabetes (OR1.2 95% CI 1.0-1.4), and higher HbA1c (OR1.1, 95% CI 1.0-1.3) were marginally independent, significant risk factors for DPN.

**Conclusions/Interpretations:**

We observed a DPN prevalence of 19.7%. Higher age, low socioeconomic status, treatment with insulin, longer duration of diabetes and poor glycemic control were risk factors for DPN.

## Introduction

Chronic peripheral sensorimotor symmetrical neuropathy (DPN) accounts for approximately 75% of the diabetic neuropathies.[1] It is defined as the presence of symptoms and/or signs of peripheral nerve dysfunction in people with diabetes mellitus (DM), after exclusion of other causes.[2] The primary symptom of DPN is loss of sensation in the toes, which extends to involve the feet and leg in a stocking distribution. Some patients complain about numbness and pain, but most frequently the disease progresses insidiously and undetected. If no action is taken, foot callus, ulceration and infection might develop and further turn into distressing and painful impairment. The foot ulcers among diabetic patients are mostly of neuropathic origin, and therefore eminently preventable.[3] Up to 85% of amputations among diabetic patients are preceded by foot ulcers.[4] The prevalence of DPN varies in the literature from 5-100%, which may reflect the different diagnostic criteria and diverse study populations.[5] Age, duration of diabetes and poor glycemic control are recognized as risk factors for DPN, while cigarette smoking, retinopathy, hypertension, obesity, hyperlipidaemia and microalbuminuria has been pointed out as potential risk indicators.[1]

It has been reported that the risk of diabetes related amputations and the prevalence of diabetic foot ulcers is significantly lower in Asians compared to Europeans in the U.K.[6–8] The reduced risk in Asians was found to be related to the lower levels of peripheral arterial disease (PAD) and DPN, but the reason is not fully understood. Ethnic differences and unknown risk factors in different populations have been proposed. There are a few DPN studies from the South-Asian region, where the prevalence of type 2 diabetes and its complications are predicted to rise extensively in the coming years.[9] To the best of our knowledge, there are no published data regarding the DPN prevalence in Bangladesh where the prevalence of type 2 diabetes has been reported to be 8,1% in the urban areas.[10] The aim of this study was to estimate the prevalence of DPN and to identify its risk factors in type 2 diabetic patients attending the Bangladesh Institute of Research and Rehabilitation in Diabetes, Endocrine and Metabolic Disorders (BIRDEM), with a view to provide necessary data to identify differential risk factors, which may ensure improved preventive measures and care for diabetic patients.

## Materials and Methods

We carried out a cross-sectional study in the outpatient department (OPD) of BIRDEM hospital, Dhaka, Bangladesh from July 2006 to September 2006. BIRDEM is a 550-bed general tertiary level hospital with the most modern disciplines. The OPD is mainly dedicated to diabetic patients, the turnover is approximately 3000 patients, including 60 to 70 new patients, per day.[11] All the subjects for investigation were recruited from BIRDEM. The inclusion criterion was type 2 diabetic outpatients diagnosed in accordance with the World Health Organization (WHO) criteria within 5-11 years prior to the investigation. The exclusion criteria were any known rheumatic disease, vitamin B_12_ deficiency, alcoholism, drug-abuse, hypothyroidism, paraneoplastic disorders, cerebral vascular disease, Parkinsonism, uremia and acute or chronic musculoskeletal disorders. The rationale behind selecting subjects with long duration was to include sufficient number of patients with DPN so that determinants of DPN can be identified.

The patient list for the OPD was made three days prior to the doctor appointment. The list was then distributed among 10 investigation rooms with two to six doctors in each room. In order to match the investigating team members' (the researcher and two assistants) credentials, one, two or three examination room(s), depending on the number of doctors attending, was randomly drawn every day. The doctors were well informed of the research objectives, procedures and the inclusion, exclusion criteria. The doctors informed and requested the appropriate patients to stay in contact with the research department after the initial examination. The patients were informed about their right to withdraw and restrict their data from analysis at any stage. Informed consent was secured prior to inclusion in the study, which was carried out according to the Helsinki declaration. The Ethical Committee of Medical Research in Norway and the BIRDEM hospital approved the protocol.

A total of 303 patients were examined. One patient withdrew from the study and seven patients were excluded due to complications related to stroke, ankle and low back operations. One patient was excluded due to diabetes duration of less than four years. Subsequently, a total of 294 patients remained for analyses.

The laboratory analyses were done at the BIRDEM hospital. Eight ml whole blood was drawn from each patient, and urine was collected in a glass test tube (6ml). Glycosylated hemoglobin (HbA1c) was analyzed by high-performance liquid chromatography (HPLC). Good glucose control was defined as HbA1c th ≤ 7.0.[12] The total cholesterol (TC) was measured using conventional laboratory techniques. The urine creatinine level was measured by the Alkaline Picrate (Hitachi 704 Japan) method in the biochemical laboratory, and urine albumin by the Nephelometry (Bn-2 Nephelometer) method in the immunology laboratory.[13,14] The detection limit for albumin was 11.6 mg/l. The urinary albumin-to-creatinine ratio (UACR) was calculated. A value < 2.5 mg/mmol was defined as normal, 2.5-30 mg/mmol as microalbuminuria and >30 mg/mmol as manifest proteinuria.[15]

Information regarding diagnosis, registration date, medication, height and the present day's blood pressure (BP) and weight was collected from the patients' medical record book. High BP was defined as systolic blood pressure ≥ l40 mmHg or diastolic pressure ≥ 90 mmHg.[16] The body mass index (BMI) was calculated according to the formula BMI = weight/height^2^ (kg/m^2^). Waist and hip circumference were measured with a non-stretchable measuring tape. Waist girth was measured through the midway between the lower border of the ribs and the iliac crest on the mid-axillary line. Hip circumference was measured to the nearest centimeter at the greatest protrusion of the buttocks just below the iliac crest. Both measurements were done with the patient standing and breathing normal. A WHR > 0.90 in men and > 0.80 in women was defined as abdominal obesity.[10]

A structured questionnaire with clear and simple questions was made for this study. An academic in the field of community medicine translated the questionnaire into Bengali. It was used to prevent any language misinterpretation between the researcher and the participants. The Bengali version was pilot tested on five patients fulfilling the inclusion criteria. There were no remarks or misunderstandings, and no changes were made.

Information regarding demographic and socioeconomic factors (age, gender, average monthly income per family member and years of education) and lifestyle characteristics (smoking history, protein intake) was obtained by interview. The Neuropathy Symptom Score (NSS) was recorded by interview following the standard guidelines.[17] The NSS consists of five questions; each assigning points in order to calculate the total symptom score. The total maximum abnormal symptom score was 9 points.

- Burning/numbness/tingling (2p) or Fatigue/ Cramping /Aching feelings (1 p) in the lower extremity
- Symptoms present in the feet(2p) or in the calf (1p)
- Nocturnal exacerbation of the symptoms (2p) or present equally at day and night (1p)
- The symptoms awake the patient from sleep (1p)
- Walking (2p) or standing (1p) maneuvers reduce the symptoms

The Neuropathy Disability Score (NDS) consists of four clinical tests on both feet.[17] The procedure was explained and the tests applied on the patient's hand prior to the examination. The patient had to close the eyes during the examination. Each test was assessed with points to calculate the total disability score. The total maximum abnormal disability score was 10 points.

- *Achilles tendon reflex:* The broad end of the reflex hammer (Babinski) was applied at the Achilles tendon. Jerk with reinforcement (1p), no jerk (2p).
- *Vibration perception:* A 128-Hz vibrating fork (Hartmann C128) was applied longitudinally on the first toe three times with at least one false application (not-vibrating fork). The patient was required to tell which application that was vibrating or not. Two of three right responds were set to be a correct answer (0p), two of three wrong responds were an incorrect answer (1p).
- *Thermal sensation (cold sponge):* One cold and one room temperature sponge was applied on the dorsum of the foot. The patients were required to tell which application was cold or normal, correct answer (0p), incorrect answer (1p).
- *Tactile sensation (pin-prick):* The reverse end of the turning fork and tendon hammer, sharp and dull respectively, was applied at the cuticle of the 1^st^ toe. The patients were required to tell which application was sharp or dull, correct answer (0p), incorrect answer (1p).

A total symptom score of 3-4 points was considered as mild symptoms, 5-6 points as moderate symptoms and 7-9 points as severe symptoms. A total disability score 3-5 points was considered mild disability, 6-8 points as moderate disability and 9-10 points as severe disability. The minimum acceptable criteria for diagnosis of DPN were moderate disability, with or without symptoms, or mild disability with moderate symptoms. Mild disability alone or with mild symptoms was not considered adequate to make a diagnosis of DPN.[17]

The data was entered in the SPSS 14.0 for Windows software. The variables age, diabetes duration and income were categorized. Descriptive statistics were used to identify DPN prevalence, determined in simple percentages. For comparison of baseline variables between the groups, the Chi-Square (χ^2^) or Fisher's exact test was preformed for categorical data, the t-test for normally distributed continuous data and the Mann-Whitney test for non-normally distributed continuous data. Spearman correlation was used to assess the relationship between variables of interest.

Bivariate and multivariate logistic regression analyses were performed to identify factors associated with DPN and adjust for potential confounding factors. Odds ratios (OR) with 95 % confidence interval (CI) were provided. Statistical significance was set at p < 0.05. All tests performed were two tailed.

## Results

There were 155 (52.7%) female and 139 (47.3%) male subjects [Table 1]. The mean age was 50.8 ± 10.6 years, females being significantly younger (48.7 ± 10.7) than men (53.1 ± 9.9) (p<0.001). The mean duration of diabetes was 7.0 ± 1.8 years [Table 1], and was similar in males and females. The overall prevalence of DPN in this population was 19.7 %, and fairly comparable for male (n=29, 20.9%) and female (n=29, 18.7 %) patients.

**Table 1:** Demographics and clinical variables related to diabetic peripheral neuropathy

Characteristics	*n*	Total sample (*n* = 294)	DPN subjects (*n* = 58)	Non DPN subjects (*n* = 236)	*P*-value for the difference
		Mean ± SD			*t*-test
Age (years)	293	50.8 ± 10.6	55.1 ± 10.5	49.7 ± 10.3	<0.001
Diabetes duration (years)	294	7.0 ± 1.8	7.7 ± 1.9	6.9 ± 1.8	0.05
Waist/hip ratio	289	0.93 ± 0.06	0.9333 ± 0.0599	0.9324 ± 0.0603	NS
BMI (kg/m^2^)	294	24.43 ± 3.35	24.16 ± 3.60	24.50 ± 3.29	NS
HbA1c (%)	293	8.75 ± 2.20	9.54 ± 2.52	8.56 ± 2.08	<0.01
Total cholesterol (mg/dl)	293	190.45 ± 31.83	191.63 ± 34.29	190.17 ± 31.27	NS
		Median (Interquartile Range)			Mann-Whitney test
			
Monthly income pr family member (TK)	294	1345.24 (1866.67)	1081.17 (1535.71)	1408.33 (2024.68)	<0.02
Education (years)	294	6.5 (12)	5.0 (12)	8.0 (12)	NS
Systolic BP (mmHg)	294	120 (10)	130 (20)	120 (10)	NS
Diastolic BP (mmHg)	294	80 (0)	80 (0)	80 (0)	NS
	287	0.00 (0)	0.00 (15.3)	0.00 (0)	<0.05**
		% (n)			Pearson Chi-square test
			
Insulin treatment	120	40.8 (120)	60.3 (35)	36.0 (85)	<0.001
Oral treatment	153	52.0 (153)	36.2 (21)	55.9 (132)	<0.01
No medication	21	7.2 (21)	3.5 (2)	8.1 (19)	NS*
Low protein intake	61	20.7 (61)	34.5 (20)	17.4 (41)	<0.01
Medium protein intake	146	49.7 (146)	44.8 (26)	50.8 (120)	NS
High protein intake	87	29.6 (87)	20.7 (12)	31.8 (75)	NS
Never smoked	217	73.8 (217)	65.5 (38)	75.8 (179)	NS
Ex-smoker	44	15.0 (44)	19.0 (11)	14.0 (33)	NS
Smoker	33	11.2 (33)	15.5 (9)	10.2 (24)	NS

* Fisher's Exact Test;

** Although the medians in the two groups are equal, there is a subgroup in the DPN patients with high values that accounts for the significant statistical difference

An increasing trend in prevalence of DPN with increasing age was observed from 11.1% in those aged 23-40 years to 32.3% in those aged 60-80 years (OR 3.8, 95% CI 1.4-10.4) [Table 2]. The prevalence of DPN increased steadily with increasing duration of diabetes (OR1.2, 95% CI 1.0-1.4) [Table 2], from 14.1% in those diagnosed five years prior to the investigation to 27.8 % in those having 9-11 years duration of diabetes. The prevalence rate also differed following the treatment procedures for diabetes. The prevalence of DPN was 13.7% in the oral antidiabetic treated group, compared to 29.2 % in the insulin treated group (OR 2.6, 95% CI 1.4-4.7) [Table 2].

**Table 2:** Univariate analysis of risk factors for diabetic peripheral neuropathy with 95% CI

Variable	*n*	*P*-values for the differences	*P*-value for the specific range	Odds ratio (95% CI)
Female	155	NS		1.0
Male	139		NS	1.1 (0.65-2.04)
Age <40 years	54	0,014		1.0
Age 41-59 years	172		NS	1.8 (0.70-4.48)
Age >60 years	61		<0.01	3.8 (1.40-10.38)
Diabetes duration (continuous)	294	0.04	0.04	1.2 (1.01-1.36)
Income >3000 TK	63	0.053		1.0
Income 801-2 999 TK	145		<0.05	2.6 (1.01-6.49)
Income <800 TK	79		<0.02	3.3 (1.25-8.83)
High protein intake	87	0.015		1.0
Middle protein intake	146		NS	1.3 (0.65-2.85)
Low protein intake	61		<0.01	3.1 (1.36-6.86)
Overweight (WHR)	255	0.036		1.0
Normal (WHR)	32		<0.05	2.3 (1.06-5.18)
HbA1c (continuous)	293	0.003	<0.01	1.2 (1.06-1.37)
Oral treatment	150	0.004		1.0
No medication (diet)	18		NS	0.7 (0.14-3.05)
Insulin treatment	119		<0.01	2.6 (1.41-4.74)
Normal BP	220	0,070		1.0
High BP	74		0.07	1.8 (0.95-3.30)
UACR <2, 5 mg/mmol	234	0,063		1.0
UACR 2,5-3, 0 mg/mmol	26		NS	1.1 (0.40-3.15)
UACR >3, 0 mg/mmol	27		<0.02	2.8 (1.18-6.48)

There was no significant correlation between the treatment procedure and age, or between the treatment procedure and duration of diabetes. A significant correlation was detected between the treatment procedure and income (rSp = 0.00135841; p< 0.001). The prevalence increased with decreasing income from 9.4% in the group earning ≥ 3000 TK per month to 25.3% in the group earning ≤ 800 TK per month (OR 3.3, 95% CI 1.3-8.8), and with decreasing protein intake from 13.8 % in the group having a high protein intake compared to 32.8% the group having a low protein intake (OR 3.1, 95% CI 1.4-6.9) [Table 2]. There was a significant positive correlation between protein intake and HbA1c (rSp = 0.00052095; p< 0.01), and between the protein intake and income (rSp = 0.000001; p< 0.001).

Age ≥ 60 years (OR 4.2, 95% CI 1.4-12.3), treated with insulin (OR 2.0, 95% CI 1.0-4.0), low/normal WHR (OR 3.7, 95% CI 1.5-9.3), and income ≤ 800 TK (OR 3.2, 95% CI 1.1-9.4), remained as statistically significant risk factors, whereas duration of diabetes (OR1.2, 95% CI 1.0-1.4), and HbA1c (OR 1.1, 95% CI 1.0-1.3), remained as borderline, statistically significant risk factors for DPN after controlling for potential confounding factors included in the multivariate logistic regression model [Table 3].

**Table 3:** Odds ratio and 95% CI of diabetic peripheral neuropathy by the following risk factors in a multivariate model

Factors	*P*-value for the entire variable	*P*-value for the specific range	Odds Ratio (95% CI)
Age <40 years	0.030		1.0
Age 41-59 years		NS	2.3 (0.83-6.22)
Age >60 years		<0.01	4.2 (1.41-12.28)
Oral treatment	0.063		1.0
No medication (diet)		NS	0.3 (0.03-2.82)
Insulin treatment		<0.05	2.0 (1.00-4.03)
Overweight (WHR)	0.006	<0.01	1.0
Low/Normal (WHR)			3.7 (1.47-9.34)
Income >3000 TK	0.093		1.0
Income 801-2 999 TK		<0.05	2.8 (1.01-7.67)
Income <800 TK		<0.05	3.2 (1.09-9.42)
Diabetes duration (continuous)	0.07	0.07	1.2 (0.99-1.40)
HbA1c (continuous)	0.09	0.09	1.1 (0.98-1.31)

## Discussion

The overall prevalence of DPN in this study was 19.7 %. It is lower compared to European studies using similar diagnostic criteria,[12,17,18] which have reported an overall DPN prevalence of 32.1% (mean age: 63 years, mean duration of diabetes: six years);[17] 35.4% (mean age: 61.3 years, mean duration of diabetes: 9.7 years)[18] and 60.0 % (mean age: 57.2 ± 10.3, mean duration of diabetes: 8.52 ± 7.13 years)[12] among type 2 diabetic hospital outpatients. The prevalence rate in our study was similar to the prevalence rate found in a study from a diabetic center in India, reporting a neuropathy prevalence of 19.1% among type 2 diabetic outpatients (mean age in the DPN-group: 62 ± 8 years, mean duration of diabetes: 12 ± 8 years).[19] As compared to the results from India, the diabetes complication in Bangladeshi subjects emerged earlier, both with respect to the age of the patient and duration of diabetes. However, the diagnostic criteria used in the study from India differ from ours and therefore no firm conclusions can be made.

We used similar diagnostic criteria as studies from the U.K. showing a lower DPN prevalence among type 2 diabetic South-Asian patients compared with European patients living in the U.K. even after adjusting for age.[6,7] However, the observed lower DPN prevalence rate in our study compared to the European studies may be explained by the duration of diabetes in the study population. The mean age of our subjects was 50.8 ± 10.55 years, which may confirm that the diabetes population in this part of the world is relatively young compared to the West.[10,20]

The results from the multiple logistic regression analysis revealed that age and duration of diabetes[1,12,17–19,21,22] are statistically significant risk factors for DPN. Duration of diabetes was only a marginally, statistically significant risk factor in our study, and may be explained by possible late diagnosis. We found no difference in the DPN rate between the genders, which also has been confirmed by others.[18,19,16,21] Our figures showed a numerical higher occurrence of DPN among smokers and patients with high BP, hypercholesterolemia and potential microalbuminuric /proteinuric, but like others[16,12] we could not identify them as statistically significant risk factors. This is in contrast with other reports,[21,23,24] and may have been due to the limited sample size.

We found a significant correlation between the treatment modality and income, and between the treatment modality and DPN. This is in agreement with other studies showing that subjects treated with insulin are at increased risk for DPN.[21,19,25] The examination for this association is not readily apparent, but it can cannot be excluded that it is due to poorer glycemic control prior to the initiation of insulin treatment.[25] The association between DPN and insulin treatment may also be a possible consequence of the welfare system provided by the BIRDEM hospital. Insulin is supplied free or at a subsidized cost to those who can not afford to pay, which may have resulted in more insulin treatment among the poorer patients. We found low income and low/normal WHR to be significant risk factors for DPN, in addition to a significant correlation between protein intake and income and between protein intake and HbA1c. This is in agreement with the findings from India indicating that poor socioeconomic background contributes to diabetic foot complications.[20]

Possible explanations for the phenomenon could be that poor people are less likely to use health services,[26] which might result in late diagnosis and uncontrolled DM. Despite of the importance of DPN diagnosis, we lack a simple accurate and readily reproducible method of measuring DPN. Population, recruitment, diagnostic criteria and modes of investigation are factors that may influence the differential results reported in various studies. We have used similar diagnostic procedures as those used in Young's study from the U.K. involving 6487 type 2 diabetic patients.[17] The method provides simple clinical criteria without referring to electrodiagnostic studies, as highly sophisticated and expensive procedures are less suitable to put into practice in developing countries like Bangladesh.

Our data suggest that the prevalence of DPN increases with age, poverty and type of treatment provided and subtly by the duration of diabetes and poor glycemic control. Our results were generated from a relatively small study in diabetic population with duration of 5-11 years prior to investigation, and the DPN prevalence rate should therefore be interpreted with some caution. However, the findings of early age for the onset of diabetes and its complication in Bangladesh, and correlation insulin treatment with increased risk for DPN deserve further attention. The data of DPN from the South Asian population, where the prevalence of type 2 diabetes is likely to increase substantially in the near future, is scarce. Therefore, the data on DPN from this population is vital to improve the preventive measures and the quality of care related to foot complication among type 2 diabetic patients.

*Duality of interest* The authors declare that there is no duality of interest for this study.
